# Hydrogen Sulfide Inhibits A2A Adenosine Receptor Agonist Induced β-Amyloid Production in SH-SY5Y Neuroblastoma Cells via a cAMP Dependent Pathway

**DOI:** 10.1371/journal.pone.0088508

**Published:** 2014-02-11

**Authors:** Bhushan Vijay Nagpure, Jin-Song Bian

**Affiliations:** Department of Pharmacology, Yong Loo Lin School of Medicine, National University of Singapore, Singapore; University of Iowa, United States of America

## Abstract

Alzheimer's disease (AD) is the leading cause of senile dementia in today's society. Its debilitating symptoms are manifested by disturbances in many important brain functions, which are influenced by adenosine. Hence, adenosinergic system is considered as a potential therapeutic target in AD treatment. In the present study, we found that sodium hydrosulfide (NaHS, an H_2_S donor, 100 µM) attenuated HENECA (a selective A2A receptor agonist, 10–200 nM) induced β-amyloid (1–42) (Aβ42) production in SH-SY5Y cells. NaHS also interfered with HENECA-stimulated production and post-translational modification of amyloid precursor protein (APP) by inhibiting its maturation. Measurement of the C-terminal APP fragments generated from its enzymatic cleavage by β-site amyloid precursor protein cleaving enzyme 1 (BACE1) showed that NaHS did not have any significant effect on β-secretase activity. However, the direct measurements of HENECA-elevated γ-secretase activity and mRNA expressions of presenilins suggested that the suppression of Aβ42 production in NaHS pretreated cells was mediated by inhibiting γ-secretase. NaHS induced reductions were accompanied by similar decreases in intracellular cAMP levels and phosphorylation of cAMP responsive element binding protein (CREB). NaHS significantly reduced the elevated cAMP and Aβ42 production caused by forskolin (an adenylyl cyclase, AC agonist) alone or forskolin in combination with IBMX (a phosphodiesterase inhibitor), but had no effect on those caused by IBMX alone. Moreover, pretreatment with NaHS significantly attenuated HENECA-elevated AC activity and mRNA expressions of various AC isoforms. These data suggest that NaHS may preferentially suppress AC activity when it was stimulated. In conclusion, H_2_S attenuated HENECA induced Aβ42 production in SH-SY5Y neuroblastoma cells through inhibiting γ-secretase via a cAMP dependent pathway.

## Introduction

Alzheimer's disease (AD), which is the most common neurodegenerative disease, is also the leading cause of senile dementia [1]. The prevalence of AD has been increasing exponentially from 3% among those above 65 years to almost 50% among those with age above 85 years [2]. AD has been one of the most debilitating diseases of the current century, with profound economic, political and social consequences.

The classical neuro-pathological hallmarks of AD include accumulation of senile plaques in the brain, neuro-fibrillary tangles, synaptic loss and neuronal death [3], [4]. Senile plaques consist mainly of a 39–42 amino acid long β-amyloid (Aβ) peptide, generated from a larger transmembrane amyloid precursor protein (APP) [5]. In non-amyloidogenic pathway, APP is cleaved within the Aβ domain by α- secretase, releasing soluble APP (sAPPα) extracellularly. On the other hand, poorly soluble amyloidogenic Aβ is derived from sequential cleavage of APP by β- and γ-secretases [6]. Above-mentioned and other evidences strongly support that APP synthesis and proteolysis are critical events in AD pathogenesis. Hence drugs targeting these processes are likely to be beneficial for the prevention and treatment of AD.

Neurodegeneration in AD leads to many neuropsychiatric symptoms by altering various essential brain functions such as cognition, memory, neuronal maturation, neuronal damage and regeneration. These brain functions are influenced by adenosine, an ubiquitously present purine ribonucleoside [7], [8]. Acting as an endogenous ligand, it exerts its functions via four G-protein coupled receptors (A1A, A2A, A2B and A3). While Gi/o coupled A1A and A3 receptor subtypes inhibit adenylyl cyclase (AC), A2A and A2B subtypes stimulate AC through Gs GTP-binding protein [9]. Adenosine plays a very important role in central nervous system (CNS) acting as a neuromodulator by regulating synaptic transmission, synaptic plasticity and neuronal excitability [10]. Indeed, adenosine signaling has been considered as a target for therapeutic intervention in major neurodegenerative diseases [11] since its definitive role has been highlighted in Alzheimer's disease [12], Parkinson's disease (PD) [13] and Huntington's disease [14]. The metabolism of adenosine in the brain is critical in the context of AD pathology. Under normal physiological conditions, the concentration of extracellular adenosine is moderated by various factors including enzymes such as adenosine kinase [15]. Although pathogenesis of AD is still unclear, there are numerous evidences suggesting that oxidative stress in the brain of AD patients results in neuronal trauma and degeneration [16], [17]. The stress and trauma initiate cascade of pathological events disrupting delicate balance of adenosine nucleotides and nucleosides altering ATP-ADP ratio [18], [19]. The resultant increase in ADP level leads to enhanced adenylate kinase enzyme activity which in turn augments AMP concentration to keep ADP-AMP ratio constant [20]. The elevated AMP undergoes hydrolysis to generate more adenosine [21]. This intracellular adenosine is released outside the cell resulting in increased extracellular concentration of adenosine [18].

Hydrogen sulfide (H_2_S) has been reported as an important modulator in multiple physiological systems including adult CNS [22], [23], [24]. The role of H_2_S in intracellular calcium ([Ca^2+^]_i_) homeostasis in neurons and glial cells is perhaps the most important one as it regulates synaptic activity and plasticity. H_2_S upregulates [Ca^2+^]_i_ in neurons and glia by increasing its influx through different Ca^2+^ channels and its receptors and by releasing calcium from [Ca^2+^]_i_ stores [24], [25], [26]. H_2_S was found to activate K_ATP_ channels, which apart from mediating neurotransmitter release from presynaptic neurons, also offer neuroprotection during hypoxic challenge [27], [28]. The neuromodulatory role of H_2_S is also critical as it maintains excitatory-inhibitory balance of neurotransmission [29]. γ-aminobutyric acid B receptors (GABA_B_R) which are involved in fine tuning of inhibitory neurotransmission and regulation of the release of neurotransmitters, are upregulated by H_2_S [30]. H_2_S is shown to modulate long-term potentiation in active synapses and neuron-glia interactions [24], [31]. Acting as a mediator of cell signaling, this gaseous neurotransmitter has been identified to target a number of ion channels, transcription factors and protein kinases [32]. The beneficial effects of H_2_S have already been found in cell and animal models of PD [33], neuroinflammation [34] and H_2_O_2_ induced neural injury [35]. Adding further in the knowledge, we studied the inhibitory effect of H_2_S against Aβ42 production in SH-SY5Y neuronal cells. In the present study, we have confirmed that A2A receptor stimulation enhanced γ-secretase activity which in turn resulted in increased Aβ42 production in SH-SY5Y cells. We also show that H_2_S attenuates Aβ42 synthesis via suppression of cAMP signal transduction pathway.

## Materials and Methods

### Chemicals

Sodium hydrosulfide (NaHS), forskolin, 3-isobutyl-1-methylxanthine (IBMX), SQ 22536, ZM 241385, N-[N-(3,5-difluorophenacetyl)-L-alanyl]-S-phenylglycine t-butyl ester (DAPT) and methylthiazolyl tetrazolium (MTT) were purchased from Sigma Aldrich (St. Louis, MO, USA). 2-Hexynyladenosine-5′-*N*-ethylcarboxamide (HENECA) was ordered from Abcam (Cambridge, MA, USA). NaHS, HENECA, SQ 22536 and MTT were dissolved in de-ionized water, while forskolin, ZM 241385, IBMX and DAPT were dissolved in dimethylsulfoxide (DMSO). Primary antibodies against phospho-CREB, Adenosine A2A-R and β-actin were purchased from Santa Cruz biotechnology (St. Louis, MO, USA). Monoclonal antibody against APP (clone 22C11) and polyclonal antibody against APP C-terminus were from Millipore (Temecula, CA, USA).

NaHS was used as an H_2_S donor. When NaHS is dissolved in water at neutral pH, HS^−^ is released and forms H_2_S with H^+^. This provides a solution of H_2_S at a concentration that is about 33% of the original concentration of NaHS [36].

### Cell Culture and Treatments

The SH-SY5Y human neuroblastoma cell line was obtained from the American Type Culture Collection (Manassas, VA, USA). Cells were cultured in 75 cm^2^ flasks in Dulbecco's modified Eagle's medium (DMEM) supplemented with 10% fetal bovine serum (FBS), 1% penicillin (100 U)/streptomycin (100 mg/mL), and were maintained at 37°C in an incubator under a humidified atmosphere of 95% air and 5% CO_2_. Cells were split twice a week. The cells were seeded at a density of 5×10^5^ cells/well in 6-well plates a day before the transfection. The SH-SY5Y cells were lipotransfected with pcDNA4-hAPP695swe using the Lipofectamine 2000 transfection reagent. After transfection for 24 hours, cells were treated with different chemicals mentioned above. The plasmids were a kind gift from Dr. Weihong Song, University of British Columbia, Vancouver, Canada.

For each experiment, confluent cells in 75-cm^2^ flasks were seeded onto 35-mm dishes. Cells in culture dishes were used for experiments after reaching 80–90% confluence.

### Cell Viability Assay

Cell viability was assessed by MTT reduction assay as follows. At the end of treatments (as described in results section), cells were incubated at 37°C with MTT at a final concentration of 0.5 mg/ml for 4 hours. The purple formazan formed was solubilized with 150 µL DMSO. The absorbance of the colored solution was measured at 570 nm with a reference wavelength of 630 nm using Saffire 2 microplate reader (Tecan, USA).

### Intracellular cAMP Assay

A commercially available direct cAMP enzyme immunoassay kit (Cayman Chemical, USA) was used to examine the involvement of cAMP. Briefly, cells were incubated in DMEM containing 0.5% FBS. After treatment with different drugs described in results section, the cells were lysed in 0.1 M HCl for 20 minutes. 50 µL of samples were added into a 96-well plate followed by incubation with cAMP acetylcholine esterase tracer and cAMP antiserum for 18 hours at 4°C. Each sample was developed with Ellman's reagent next day and the plate was read at a wavelength of 405 nm. cAMP concentration was calculated according to the cAMP standard and the protein was quantified by dissolving the pellets.

### Cell Fractionation and Adenylyl Cyclase (AC) Activity Assay

A cell fractionation technique was adopted from the literature [37]. AC activity was assayed as described previously [38], [39] with some modifications. The AC activity assay was performed at 37°C for 10 min in a 400 µL reaction mixture containing 1 mM ATP, 100 mM NaCl, 50 mM HEPES, 0.5 mM IBMX, 6 mM MgCl_2_, 1 µM GTP, and 20 µg of membrane protein. Reactions were stopped by addition of 0.6 mL of trichloroacetic acid (10% w/v). The accumulation of cAMP was later assayed by cAMP EIA kit (Cayman Chemical, USA).

### γ-secretase (Fluorogenic Substrate) Assay

The assay was performed as described previously in the literature [40]. Briefly, the cell lysates were centrifuged at 12000 g for 15 min. Resultant pellets were resuspended and incubated overnight at 37°C in 200 µL of assay buffer containing 10 µL fluorescent substrate of γ-secretase (Calbiochem). The fluorescence was measured with excitation wavelength set at 355 nm and emission wavelength at 440 nm.

### ELISA for Aβ42

The conditioned medium from samples was collected by centrifugation (12,000 g at 4°C for 15 min). The secreted levels of Aβ42 in conditioned medium were quantitatively measured by commercially available ELISA kit (Invitrogen, USA). Briefly, the samples were diluted by standard diluent buffer and AEBSF was added to diluted samples and standards which prevent proteolysis of Aβ peptides. The standards, controls and samples were pipetted into the antibody pre-coated wells and co-incubated with a rabbit antibody specific for the C-terminus of the 1–42 Aβ sequence. Bound rabbit antibody was detected by the use of a horseradish peroxidase-labeled anti-rabbit antibody. After washing, horseradish peroxidase-labeled anti-rabbit antibody (enzyme) was added. After washing to remove the entire unbound enzyme, a substrate solution was added, which is acted upon by the bound enzyme to produce color. The intensity of this colored product is directly proportional to the concentration of Aβ42 present in the samples. The optical density was measured at 450 nm wavelength using Saffire 2 microplate reader (Tecan, USA).

### Reverse Transcription-PCR

Two-step reverse transcription polymerase chain reaction was used to determine mRNA levels of AC1, AC3, AC8, PS1, PS2 and GAPDH in SH-SY5Y cells. Total RNA was extracted using TRIzol® extraction method (Invitrogen, Carlsbad, CA, USA). Homogenized samples were then incubated at room temperature for 10 min. Chloroform was added and tubes were shaken vigorously by hand for 15 min followed by incubation for 3 min at room temperature again. Samples were centrifuged at 12000 g for 15 min at 4°C. Colorless upper aqueous phase was transferred to a new tube containing isopropanol (prepared in nuclease free water) and incubated for 10 min at 25°C followed by centrifugation at 12000 g for 10 min at 4°C. Supernatant was discarded and RNA pellets were washed with 70% ethanol (prepared in nuclease free water). RNA concentration was determined with NanoDrop Spectrophotometer (ND-1000, NanoDrop Technology). Equal amounts of RNA samples obtained were reverse transcribed into cDNA using iScript™ cDNA synthesis kit (Bio-Rad). Reverse transcription was performed at 25°C (for 5 min), 42°C (for 30 min) and 85°C (for 5 min). The resulting cDNAs were PCR-amplified using Taq DNA polymerase kit (i-DNA Biotechnology). The specific PCR primer sequences used were as follows:

AC1 (5′-CATGACCTGCGAGGACGAT-3′ and 5′-TCCCGTTCGACATGTTTGTA-3′) [41], AC3 (5′-GTACTACACGGGACCCAGCA-3′ and 5′ –GCTCTAAGGCCACCATAGGTA-3′) [41], AC8 (5′-ACCGGCATTGAGGTAGTGAT-3′and 5′- ATGACCACTTGGAGGATGAC-3′) [41], PS1 (5′-ACAGAGTTACCTGCACCGTTGTCC-3′ and 5′-TTCCTCATCTTGCTCCAC-CACCTG) [42], PS2 (5′-AGTGAGAGA-CAGCCAGAAGCAAG-3′ and 5′-AACCCCACTACAGACATAGCGGTC-3′) [42], GADPH (5′-GCGGGGCTCTCCAGAACATCAT-3′ and 5′-GGTGTC-CAGGGGTCTTACTCC-3′) [43]

PCR conditions were set as 95°C (for 30 sec), 55°C (for 30 sec), and 72°C (for 30 sec) for 40 cycles. PCR products were separated on a 1% agarose gel and stained with ethidium bromide. The optical densities of the mRNA bands were analyzed with GelDoc-It Imaging System.

### Western Blot Assay

Cells were washed twice with ice-cold PBS after treatment and solubilized in RIPA lysis buffer (150 mM sodium chloride, 1.0% Nonidet P-40, 0.5% sodium deoxycholate, 0.1% SDS, 50 mM Tris at pH 8.0, protease and phosphatase inhibitor cocktails). The cell lysates were shaken and kept on ice for 1 hour before being subjected to centrifugation at 12,000 g at 4°C for 10 min. Supernatants were collected and denatured by SDS sample buffer. Epitopes were exposed by boiling the protein samples at 95°C for 5 min. Protein concentrations were determined with a NanoDrop Spectrophotometer (ND-1000, NanoDrop Technology). Equal amounts of the protein samples were separated by electrophoresis using a 10% sodium dodecyl sulphate-polyacrylamide (SDS/PAGE) gel and transferred onto a nitrocellulose membrane (Whatman®, Germany). After being blocked in 10% milk with TBST buffer (10 mM Tris-HCl, 120 mM NaCl, 0.1% Tween-20, pH 7.4) at room temperature for 1 hour, the membranes were incubated with primary antibodies of phospho-CREB (1∶1000), β-actin (1∶1000), A2A-R (1∶1000), APP (clone 22C11) (1∶1000) and APP C-terminus (1∶1000) at 4°C overnight. Membranes were washed three times in TBST buffer, followed by incubation with 1∶10000 dilutions of appropriate horseradish peroxidase-conjugated (HRP) anti-mouse IgG or anti-rabbit IgG at 25°C for 1 hour, and washed three times in TBST. Visualization was carried out using ECL® (plus/advanced chemi-luminescence) kit (GE healthcare, UK). The density of the bands on Western blots was quantified by Image J software.

### Statistical Analysis

Values stated are mean ± SEM of at least triplicate measurements. SPSS software for Windows was used to perform one-way analysis of variance (ANOVA) followed by a *post hoc* (Bonferroni) test for multiple group comparison. The significance level was set at *p*<0.05.

## Results

### NaHS attenuates Adenosine A2A receptor agonist (HENECA) induced Aβ42 production in SH-SY5Y cells

**Figure 1:**
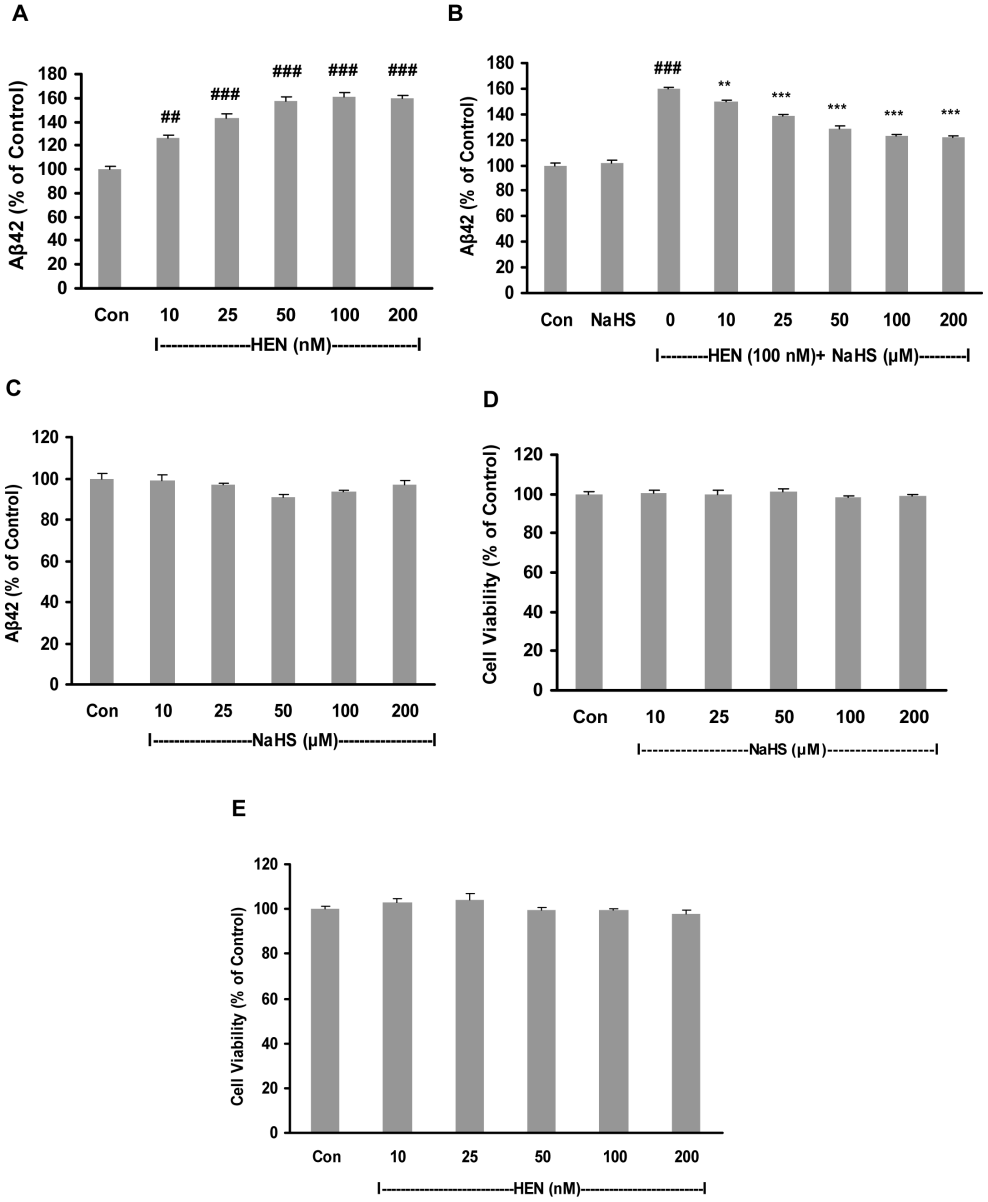
Effect of NaHS on Aβ42 production and cell viability in SH-SY5Y cells expressing APPswe. **A**: Concentration-dependent effect of HENECA (10–200 nM, 24 hours) on Aβ42 production. **B–C**: Dose-dependent effect of NaHS (10–200 µM, 12 hours) on Aβ42 formation in the presence (B) and absence (C) of HENECA (100 nM, 24 hours). **D–E**: MTT assay showing the effect of NaHS alone at 10–200 µM (D) or HENECA alone at 10–200 nM (E) on cell viability of SH-SY5Y cells. Aβ42 levels in conditioned media were measured by sandwich ELISA kit. Control values were adjusted to 100%. Data are given as means ± S.E.M, n = 6. ^##^ *p*<0.01, ^###^ *p*<0.001 vs Con group; ^**^ *p*<0.01, ***p<0.001 vs HEN group. Con, control; HEN, HENECA.

We first analyzed the effect of adenosine A2A receptor agonist, HENECA on Aβ42 production by SH-SY5Y cells. These SH-SY5Y cells were transfected with APP harboring Swedish mutation which is known to elevate secretion of Aβ42 [44]. Treatment of cells with HENECA at 10–200 nM for 24 hours increased the levels of Aβ42 in conditioned medium in a concentration-dependent manner (Fig 1A). Pretreatment with NaHS (10–200 µM) for 12 hours attenuated the stimulatory effect of HENECA (100 nM) on Aβ42 production in a dose-dependent manner (Fig 1B). However, pretreatment with NaHS (50–100 µM) alone did not reduce the basal levels of Aβ42 significantly (Fig 1C). To confirm that the observed effects of NaHS and HENECA were not due to their effects on cell viability, MTT cell viability assay were performed. MTT results showed that both NaHS (Fig 1D) and HENECA (Fig 1E) at the concentration ranges used for the experiments had no effect on cell viability.

### The effect of NaHS on HENECA-induced Aβ42 production involves cAMP/PKA/CREB pathway

**Figure 2:**
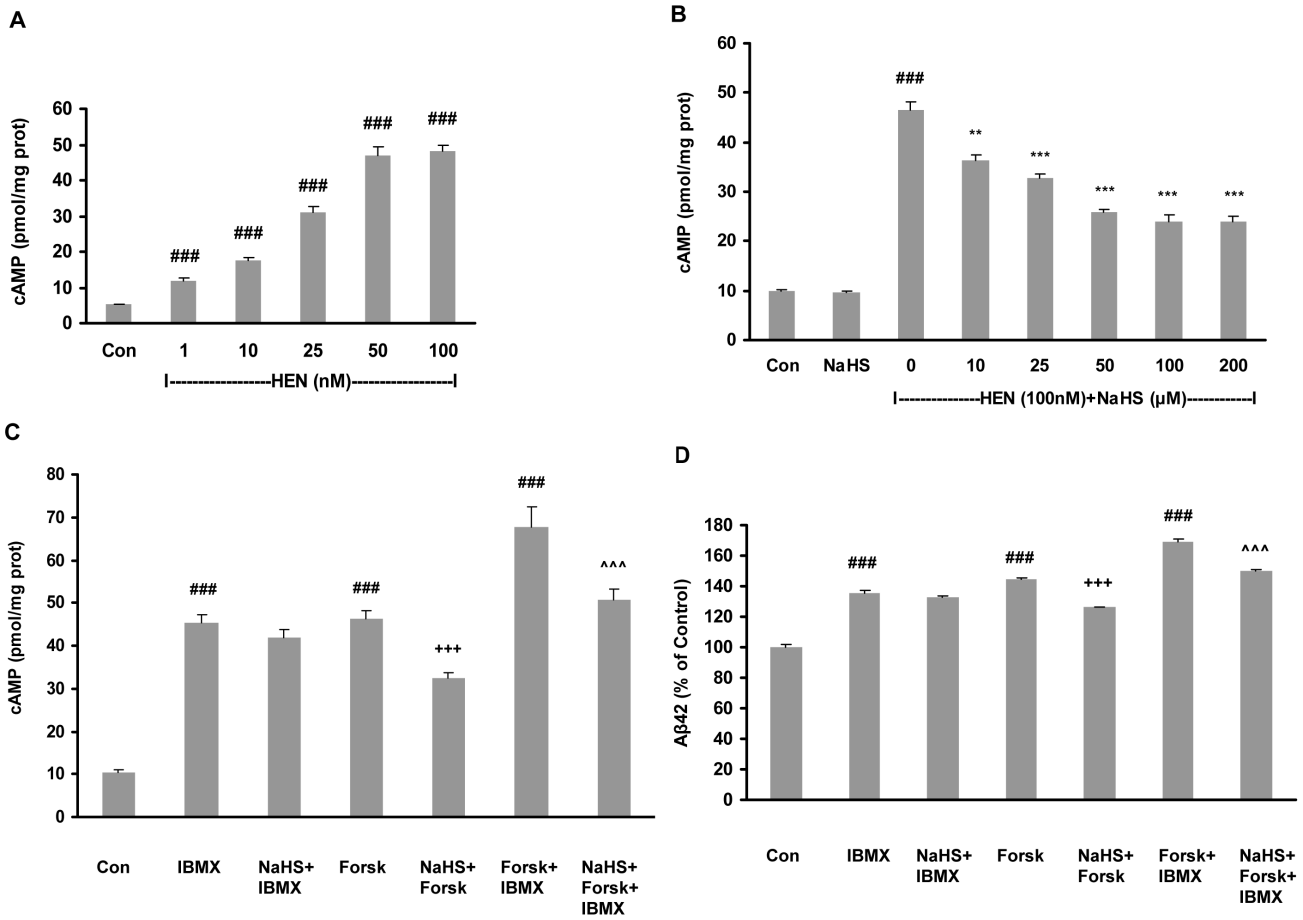
Effect of NaHS on Aβ42 production involved cAMP signaling pathway. **A**: Dose-dependent effect of HENECA on cAMP production in SH-SY5Y cells expressing APPswe. **B**: Concentration-dependent effect of NaHS (10–200 µM, 12 hours) on HENECA (100 nM, 24 hours)-stimulated cAMP upregulation. **C–D**: Effects of NaHS (100 µM) on cAMP (C) and Aβ42 production (D) in cells treated with forskolin (20 µM) and/or IBMX (100 µM). The intracellular cAMP and Aβ42 levels in conditioned media were measured by sandwich ELISA kits. Control values were adjusted to 100% for Aβ42 levels measurement. Data are given as means ± S.E.M, n = 6. ^###^ *p*<0.001 vs Con group, ^**^ *p*<0.01, ***p<0.001 vs HEN group,^+++^ *p*<0.001 vs forsk group, ^???^ *p*<0.001 vs forsk + IBMX group. Con, control; HEN, HENECA; Forsk, forskolin.

We further examined the underlying mechanism for the effect of NaHS. As shown in Fig 2A, HENECA (1–100 nM) in a dose-dependent manner increased intracellular cAMP levels. The increase in intracellular cAMP levels by HENECA was abrogated by NaHS (10–200 µM) in a concentration-dependent manner (Fig 2B). However, NaHS (100 µM) alone did not produce any significant effect on cAMP levels (Fig 2B). In an attempt to investigate whether NaHS targets the synthesis or the decomposition of cAMP, the following series of experiments was conducted. Intracellular cAMP production was elevated by forskolin, an AC agonist and IBMX, a phosphodiesterase (PDE) antagonist. As shown in Fig 2C, NaHS significantly reduced the elevated cAMP production caused by either forskolin alone or forskolin in combination with IBMX, but had no effect on those caused by IBMX alone. Similarly, NaHS treatment significantly attenuated the elevated Aβ42 production stimulated by either forskolin alone or forskolin in combination with IBMX, but had no effect on the effect caused by IBMX alone (Fig 2D).

**Figure 3:**
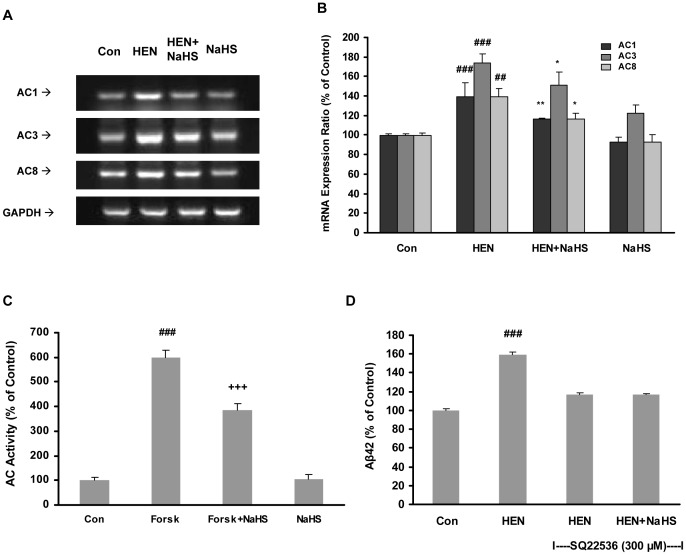
Effect of NaHS on mRNA expression of AC isoforms and AC activity. **A–B**: Representative gels (A) and histogram (B) demonstrating the effect of pretreatment with NaHS (100 µM, 12 hours) attenuated the effects of HENECA (100 nM, 24 hours) on mRNA expressions of AC isoforms. **C**: Effect of NaHS (100 µM) on AC activity stimulated by forskolin (20 µM). **D**: Effect of NaHS (100 µM) on Aβ42 production in SH-SY5Y cells preincubated with AC antagonist, SQ 22536 (300 µM). Control values were adjusted to 100%. Data are given as means ± S.E.M, n = 4–6. ^##^ *p*<0.01, ^###^ *p*<0.001 vs Con group; ^+++^ *p*<0.001 vs Forsk group, ^*^ *p*<0.05, ^**^ *p*<0.01 vs HEN group. Con, control; HEN, HENECA; Forsk, forskolin.

Gene expression studies with RT-PCR (Fig 3A and 3B) showed that HENECA upregulated mRNA expressions of all three isoforms of AC (AC1, AC3 and AC8) and NaHS pretreatment was effective in reducing their upregulated expressions. Additionally, a direct measurement of enzymatic activity of AC showed that NaHS treatment significantly suppressed the AC activity elevated by forskolin (Fig 3C). To bolster the hypothesis further, we investigated the effect of NaHS on Aβ42 generation by SH-SY5Y cells preincubated with AC antagonist SQ 22536. Neither HENECA nor NaHS was able to induce any kind of significant effect on Aβ42 production (Fig 3D) in this situation.

**Figure 4:**
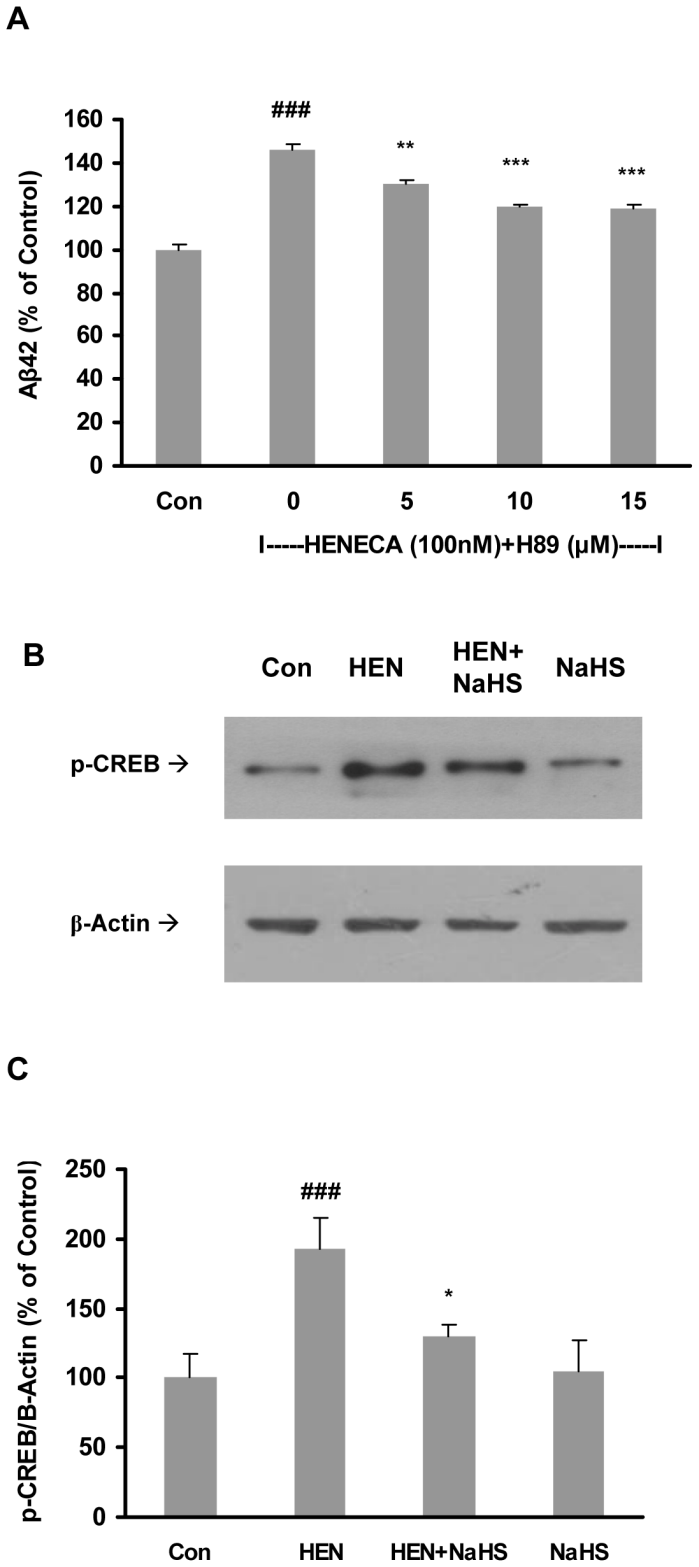
Effect of NaHS on Aβ42 production involved PKA and CREB. **A**: Effect of HENECA (100 nM) on Aβ42 formation was abolished by a PKA inhibitor, H89 (5, 10 and 15 µM). **B–C**: Representative gel (B) and histogram (C) depicting that pretreatment with NaHS (100 µM, 12 hours) attenuated the effects of HENECA (100 nM, 24 hours) on phosphorylation of CREB. Control values were adjusted to 100%. Data are given as means ± S.E.M, n = 4–6. ^###^ *p*<0.001 vs Con group; ^*^ *p*<0.05, ^**^ *p*<0.01, ***p<0.001 vs HEN group. Con, control; HEN, HENECA.

Blockade of PKA with its selective inhibitor, H-89 (5–15 µM) also dose-dependently attenuated the elevated Aβ42 induced by HENECA (Fig 4A). To examine the involvement of CREB, we determined the phosphorylation of CREB using western blotting. As shown in Fig. 4B and 4C, NaHS treatment significantly attenuated HENECA-induced phosphorylation of CREB. These results suggest that the inhibitory effect of H_2_S on Aβ42 production involves cAMP/PKA/CREB pathway.

**Figure 5:**
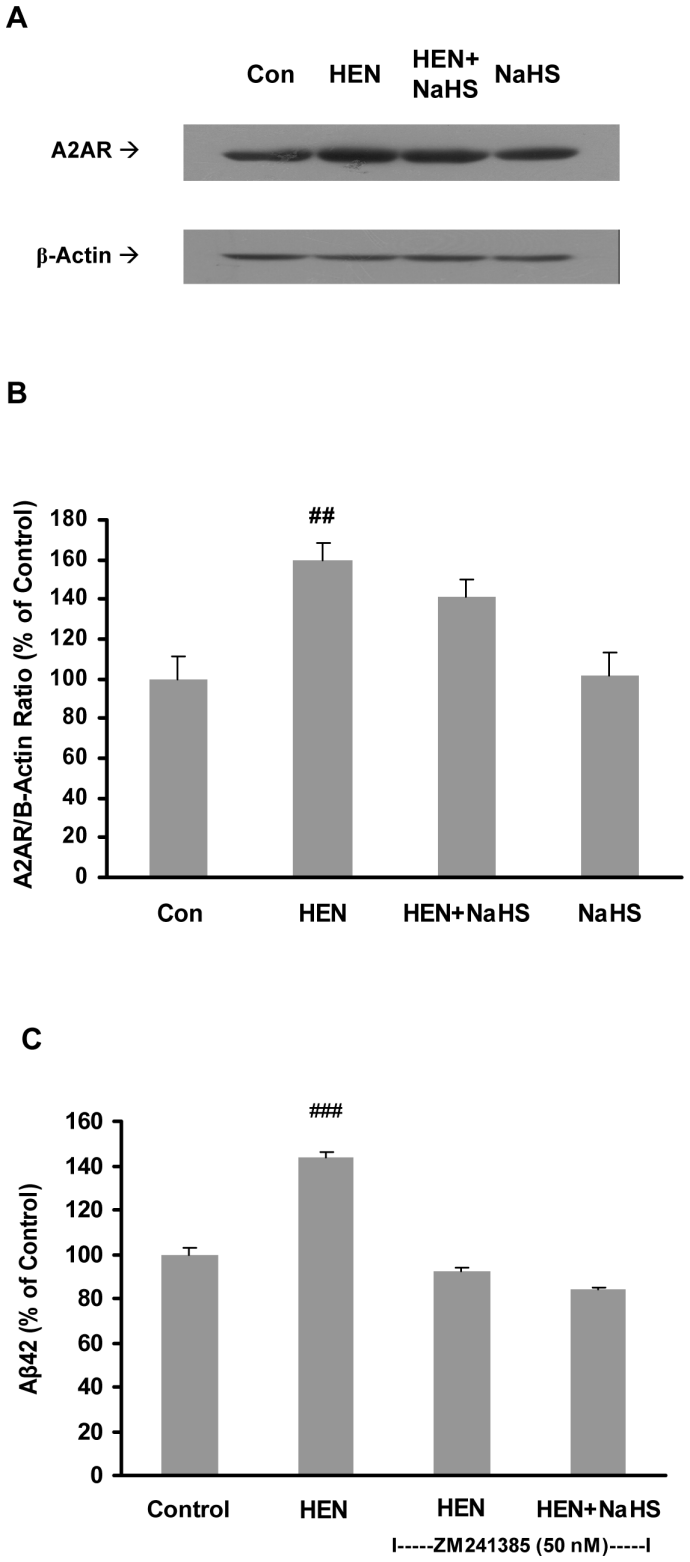
Effect of NaHS on expression of A2A receptors. **A–B**: Representative gels (A) and histogram (B) demonstrating the effect of pretreatment with NaHS (100 µM, 12 hours) did not attenuate the effects of HENECA (100 nM, 24 hours) on protein expression of A2A receptor. **C**: Effect of NaHS (100 µM) on production of Aβ42 in cells pre-treated with A2A receptor antagonist, ZM 241385 (50 nM). Control values were adjusted to 100%. Data are given as means ± S.E.M, n = 4–6. ^##^ *p*<0.01; ^###^ *p*<0.001 vs Con group. Con, control; HEN, HENECA.

Unlike its effect on AC, NaHS pretreatment was ineffective in inducing any significant effect on protein expression of A2A receptors (Fig 5A and 5B). Furthermore, we observed that Aβ42 production was not affected by either HENECA or NaHS in cells preincubated with A2A receptor antagonist, ZM 241385 (Fig 5C). Therefore, these data suggest that NaHS may preferentially suppress AC activity when it is stimulated.

### NaHS inhibits APP production and maturation

**Figure 6:**
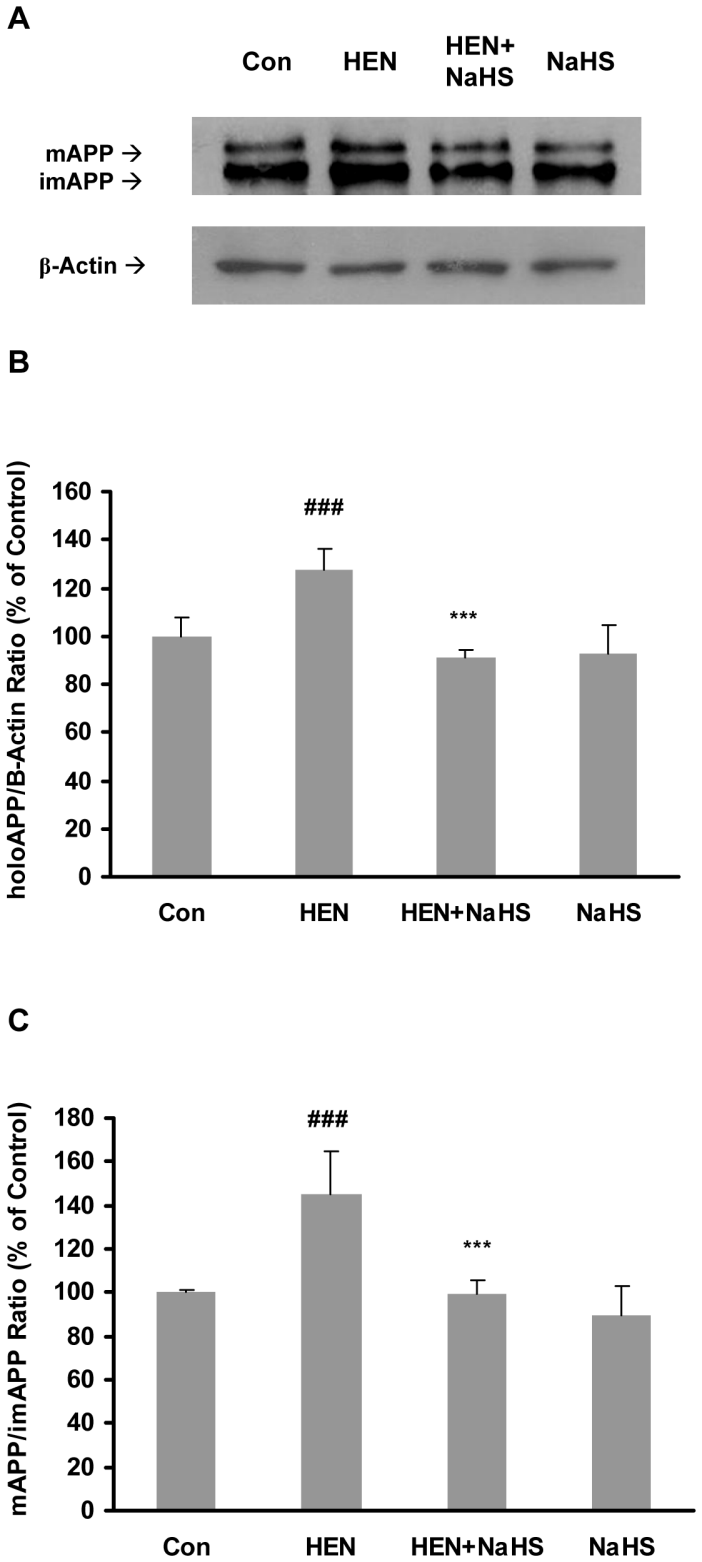
Effect of NaHS on production and maturation of APP. Representative gel (A) and quantitative analysis (B–C) showing the effects of NaHS (100 µM, 12 hours) on HENECA (100 nM, 24 hours) stimulated production (B) and maturation (C) of APP. The cell lysates were analysed by western blot technique with antibody against N-terminus of APP or β-actin. The extent of maturation of APP is shown as the ratio between mAPP to imAPP. mAPP and imAPP are represented by upper and lower bands in a blot respectively. β-actin was used as a loading control. Data are given as means ± S.E.M, n = 4. ^###^ *p*<0.001 vs Con group; ***p<0.001 vs HEN group. Con, control; HEN, HENECA.

We next sought to monitor the potential effects of H_2_S on post-translational modification (maturation), which is a major regulatory step in Aβ42 formation. During SDS-PAGE, both mature (mAPP) and immature (imAPP) forms of APP can be distinguished on the basis of their molecular weights. The immature form (*N*-glycosylated) is reported as ∼110 kD and mature form (*N*- and *O-* glycosylated) is ∼130 kD peptide [45]. As shown in Fig 6, HENECA significantly upregulated both holoprotein APP (imAPP + mAPP, Fig 6A & 6B) and the ratio of mAPP and imAPP (Fig 6A & 6C). Pretreatment with NaHS significantly abolished these effects. Thus, the effect of NaHS on HENECA-stimulated Aβ42 production is mediated by inhibition of both production and maturation of APP.

### Effect of NaHS on activities of β– and γ-secretases

**Figure 7:**
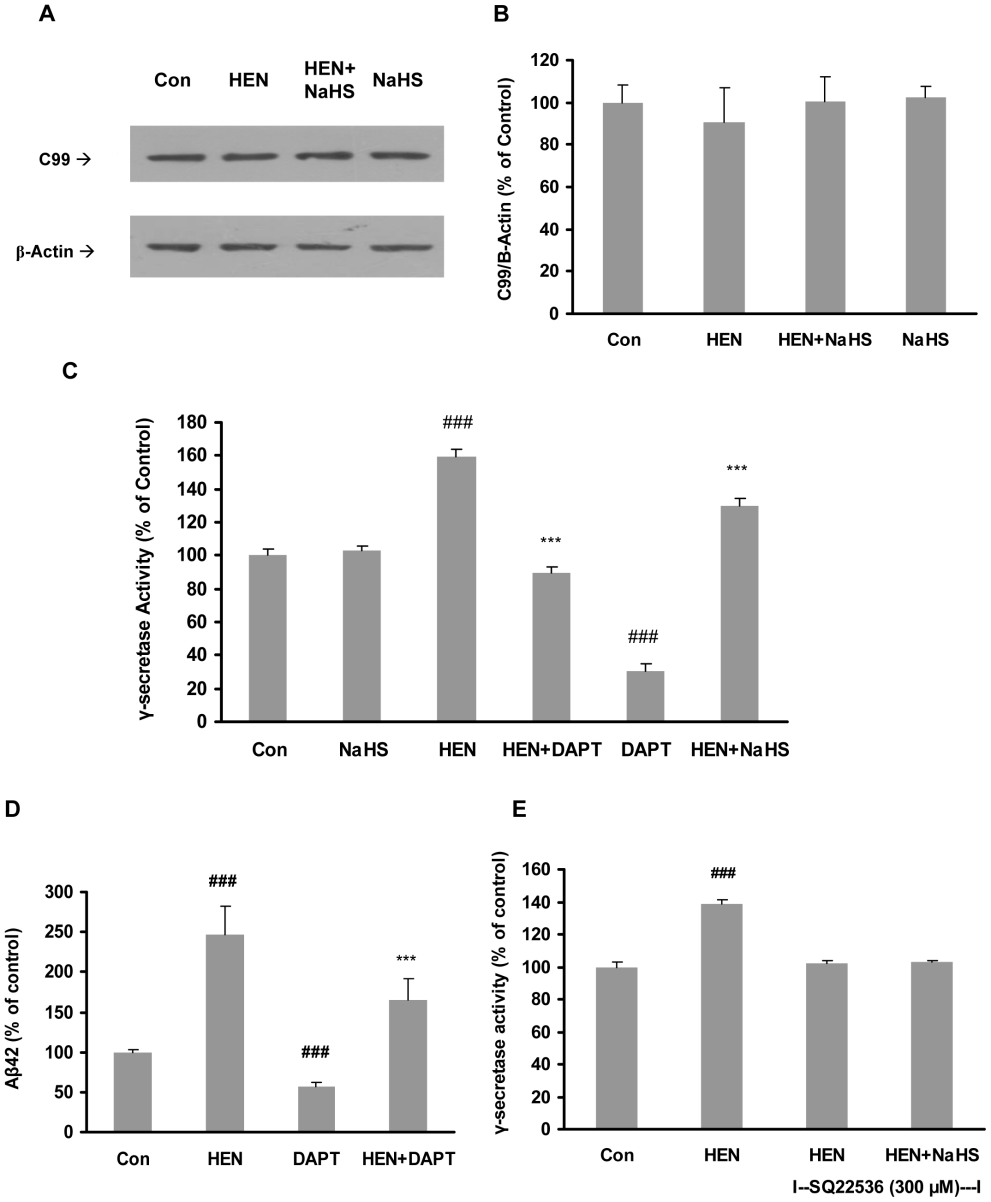
Effect of NaHS on activities of β- and γ-secretases. **A–B**: Representative gel (A) and quantitative analysis (B) showing NaHS (25–100 µM, 12 hours) and HENECA (100 nM, 24 hours) failed to affect β-CTF (C99) expression in SH-SY5Y cells. The membrane fractions were analysed by western blot with antibody against c-terminal fragment of APP (C99) or β-actin. **C–D**:Effect of NaHS (100 µM) and a γ-secretase inhibitor, DAPT (1 µM, 1 hour) on HENECA (100 nM)-stimulated γ-secretase activity (C) and Aβ42 formation (D) in SH-SY5Y cells expressing APPswe. **E**: Effect of NaHS (100 µM) on γ-secretase activity in SH-SY5Y cells preincubated with AC antagonist, SQ 22536 (300 µM). Data are given as means ± S.E.M, n = 4–6. ^###^ *p*<0.001 vs Con group; ***p<0.001 vs HEN group. Con, control; HEN, HENECA.

β –secretase cleavage of APP gives rise to production of β-C-terminal fragment, C99. Thus, the amount of C99 generated is considered as an index of β–secretase activity. As evident from Fig 7A and 7B, neither HENECA nor NaHS pretreatment (25–100 µM) was able to induce any significant change in C99 expression. This suggests that NaHS does not affect the activity of β–secretase in SH-SY5Y cells.

**Figure 8:**
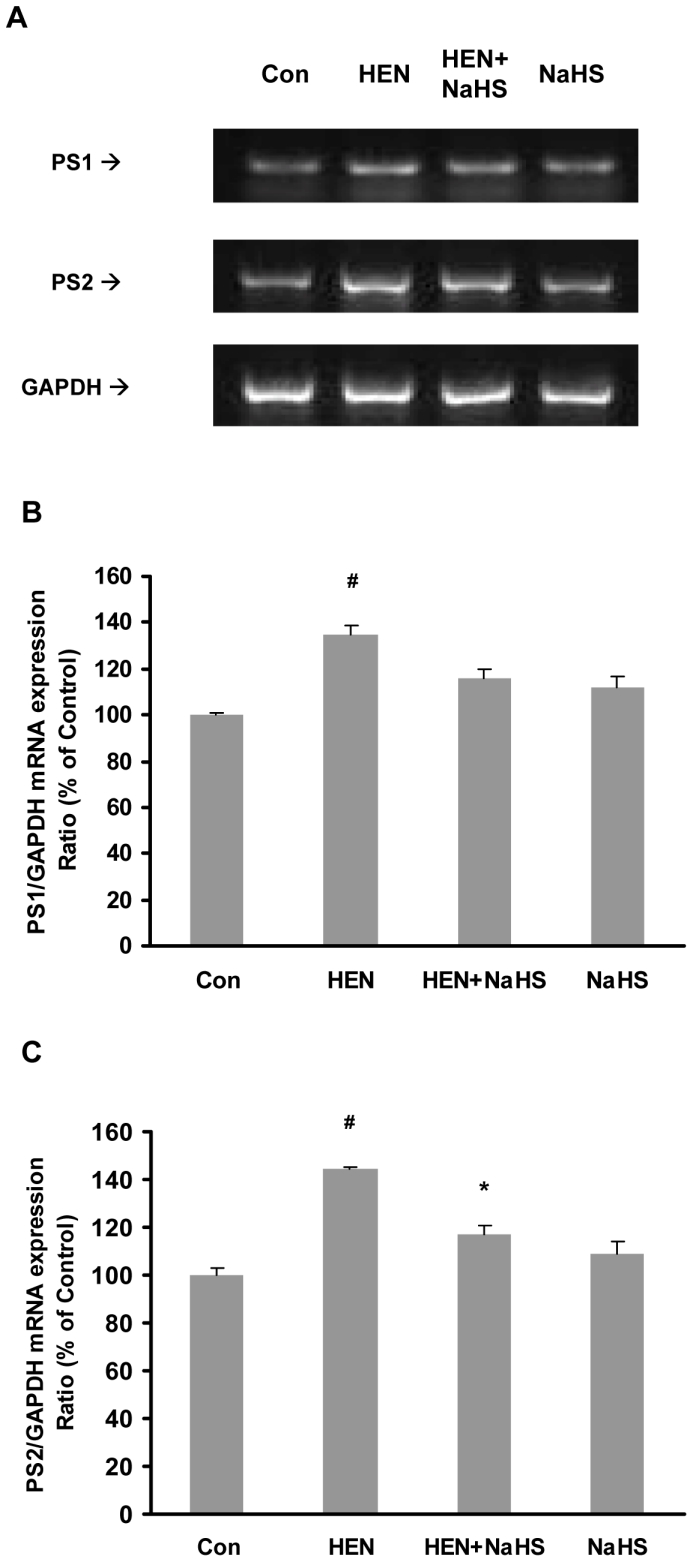
Effect of NaHS on mRNA expressions of presenilins 1 and 2. Representative gels (A) and histograms (B–C) demonstrating the effect of pretreatment with NaHS (100 µM, 12 hours) on HENECA (100 nM, 24 hours) stimulated mRNA expression of presenilins 1 and 2 respectively. Control values were adjusted to 100% for mRNA expression. Data are given as means ± S.E.M, n = 4. ^#^ *p*<0.05 vs Con group; ^*^ *p*<0.05 vs HEN group. Con, control; HEN, HENECA.

γ-secretase activity in the membrane fractions of SH-SY5Y cells was also measured. As shown in Fig 7C, HENECA significantly stimulated γ-secretase activity. Treatment with NaHS or DAPT, a γ-secretase inhibitor, significantly attenuated the effect of HENECA. It is also demonstrated that treatment of NaHS alone did not affect γ-secretase activity significantly (Fig 7C). Furthermore, DAPT reduced the production of Aβ42 in HENECA-stimulated cells (Fig 7D). In accordance with our previous result in Fig 3D, the blocking of AC with its specific inhibitor did not have any effect on γ-secretase activity (Fig 7E). We next investigated the effect of NaHS pretreatment on mRNA expressions of presenilins 1 (PS1) and 2 (PS2), which are the catalytic components of γ-secretase complex. We found that NaHS significantly suppressed HENECA-upregulated gene expression of PS2 while PS1 mRNA level was not significantly affected (Fig 8A, 8B and 8C). These results suggest that NaHS attenuates HENECA induced activation of γ-secretase. It ultimately results in lowered production of Aβ42 in the conditioned medium of SH-SY5Y cells.

## Discussion

Accumulating epidemiological, genetic and pharmaceutical studies have shown that there is a convincing role of adenosine signaling in controlling brain damage. While activation of A1A receptors is important in controlling the early events in case of brain damage, blocking of A2A receptors seems to be more important in context of the latter events. The efficiency of A1A receptors decreases as they are subjected to chronic noxious stimuli, whereas the efficiency of A2A receptors tend to remain unaffected or even increase in the similar situations [46]. It has been reported that the expression of A2A receptors is significantly increased in a transgenic mouse model of AD carrying the APP Swedish mutation [47]. The studies conducted in diverse animal models of AD showed that caffeine (a non-selective antagonist of adenosine receptors) improved memory performance in rodents with the protection against memory dysfunction [48]. It was also found that blockade of adenosine A2A receptors mimicked the neuroprotective effect of caffeine against Aβ-induced neurotoxicity [49] and prevented the development of Aβ-induced synaptotoxicity leading to memory dysfunction *in vivo* [50]. Antagonizing A2A receptor signaling, thus, seems to be one of the most promising therapeutic strategies for chronic brain pathologies such as AD [46], [51].

The present study was designed to investigate the effect of H_2_S on HENECA-induced synthesis of Aβ42 in APPswe transfected SH-SY5Y cells, an established cell model of AD. As demonstrated in our study, A2A receptor stimulation with HENECA resulted in increased production of Aβ42. Essentially, this is consistent with the findings in a previous study [47] where increased productions of both Aβ40 and Aβ42 were reported in AD transgenic mice and APPswe N2a neuronal cultures with higher adenosine receptor density. Albeit both Aβ40 and Aβ42 forms are pathological, predominantly present 42 amino-acid form of Aβ (Aβ42) peptide is readily collective and thus more pathogenic in nature [52]. We showed that NaHS inhibited HENECA-induced Aβ42 release from SH-SY5Y cells in a dose-dependent manner.

Being positively linked to AC, adenosine A2A receptor stimulation results in the increase in intracellular cAMP level. Experimental studies in both *in vitro* and *in vivo* models of AD have reported that cAMP dependent pathway is critically important in APP processing. In a study conducted with astrocytes, Lee et al. observed that cAMP signaling can increase cellular levels of APP holoprotein by stimulating APP gene expression [53]. APP protein expression and processing are shown to be increased following the elevation of cAMP level in neuronal cells [54]. Su et al. demonstrated that direct administration of PKA antagonist into the brains of transgenic mice overexpressing human APP inhibited Aβ production in the hippocampal region [55]. Our group has previously reported the inhibitory effect of H_2_S on cAMP production in various tissues like JG cells of kidney, vascular smooth tissue and heart [39], [56], [57]. Based on these reports, it was reasonable to speculate the involvement of cAMP dependent pathway in the observed effects of H_2_S.

We observed the increase in intracellular cAMP level by stimulating A2A adenosine receptors. This increase was simultaneous with stimulation in Aβ42 production. This was also true when cAMP was elevated by forskolin-induced stimulation of AC (cAMP synthesis) and IBMX-induced inhibition of phosphodiesterase (cAMP decomposition). H_2_S inhibited both intracellular cAMP production and Aβ42 production in above mentioned conditions. Curiously, there are some incongruous reports demonstrating the increase in intracellular cAMP concentration after NaHS pretreatment [58]. It seems that there are number of factors which can determine the final effect of H_2_S on intracellular cAMP levels in various cell types. The duration of treatment with and concentration of H_2_S donor could be key influences. Moreover, the presence of different isoforms of AC and/or PDE and concurrent activation/deactivation of other intracellular signaling pathways in single cell type can also alter the final effect. Nine isoforms of AC (AC 1–9) have been identified in humans till date, of which AC1, AC3 and AC8 are exclusively expressed in neurons [59]. We found that H_2_S decreased the expressions of all 3 isoforms of AC in HENECA stimulated cells. This result is consistent with the findings of recent study where NaHS attenuated upregulated protein and mRNA expression of AC isoforms and cAMP production in the striatum of morphine-dependent mice and selective μ-opioid receptor agonist treated SH-SY5Y cells [60]. Additionally, H_2_S suppressed augmented activity of AC in HENECA stimulated cells.

CREB is a primary downstream target of cAMP for secondary intracellular signal transduction. We observed that pretreatment with NaHS inhibited HENECA induced CREB phosphorylation. Furthermore, the results from experiments using a specific blocker of PKA/CREB strongly favored our speculation as it also abolished the secretion of Aβ42. Additionally, when AC was blocked with its specific inhibitor, no significant change in Aβ42 production was detected. Unlike AC isoforms, H_2_S did not have any direct inhibitory effect on A2A receptor protein expression. Furthermore, both HENECA and H_2_S failed to induce any significant effect on Aβ42 levels in the presence of A2A receptor antagonist. These results suggest that H_2_S inhibits activity and expression of AC only, thus downregulating its downstream pathway.

The production of Aβ is strictly regulated by both post-translational modifications of APP and secretases activity. The immature form of APP (imAPP) is only *N*-glycosylated and confined to endoplasmic reticulum (ER). It then undergoes maturation in the Golgi complex by *O*-glycosylation to form mature APP (mAPP). After insertion into plasma membrane, some of mAPP is reinternalized into endosomes to generate Aβ [61]. Any disruption in trafficking and maturation of APP might result into AD pathogenesis [61], [62]. It was found that PKA inhibition results in imAPP accumulation and leads to reduced Aβ42 production [55]. We found that H_2_S significantly abolished the effect of HENECA on mAPP/imAPP ratio. Furthermore, H_2_S is known to downregulate sarcoplasmic/endoplasmic reticulum calcium ATPase pump (SERCA) [63]. These findings suggest the possible alteration of APP metabolism by H_2_S at the level of ER. It is also possible that H_2_S disrupts reinternalization of mAPP into endosomes. Although inhibition of APP maturation by H_2_S suggests its accountability for the decreased Aβ42 production, the exact underlying mechanism is still elusive and needs further study.

The first proteolytic cleavage of APP is carried out by an aspartyl protease named BACE1, the key rate limiting enzyme of Aβ production. We could not find any evidence suggesting that H_2_S inhibits BACE1 as the expression of β -CTF, C99 was unaffected. However, Zhang et al. have reported that H_2_S downregulated BACE-1 expression and Aβ42 production in unstimulated rat pheochromocytoma PC12 cells [64]. The apparent inconsistencies between these two reports may be explained on the basis of differences in experimental parameters such as cell type, APPswe transfection and duration of NaHS treatment. More importantly, direct β- activity was not measured in our study.

On the other hand, H_2_S seems to affect the proteolytic processing of APP by γ-secretase. It is a high-molecular weight complex consisting of at least four components: presenilins (PS1 and PS2), nicastrin, anterior pharynx defective 1 (APH-1) and presenilin enhancer 2 (PEN-2) [65]. Each of these components has its own physiological function and are necessary together for full proteolytic activity of the complex. The relevance of γ-secretase complex to AD pathology became irrefutable when PS1 and PS2 were identified as major pathological genes in familial AD [66]. In the current study, we confirmed that up-regulated cAMP level and CREB phosphorylation by adenosine A2A receptor stimulation resulted in enhanced γ-secretase activity. We found that H_2_S suppressed HENECA-elevated γ-secretase activity as evident from direct measurement. Further, we also found that H_2_S significantly attenuated the HENECA- stimulated gene expression of major catalytic subunit of γ-secretase complex, PS2. This specific alteration of only PS2 mRNA level and not PS1's may be explained by the probability of the existence of separated regulatory systems controlling the expression of PS1 and PS2 genes in human neural cells [42].

**Figure 9:**
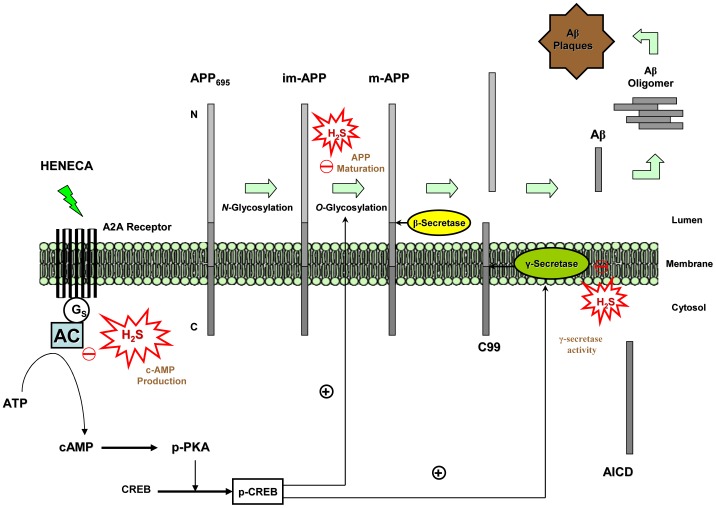
Schematic diagram showing the inhibitory effect of H_2_S on HENECA induced Aβ generation in SH-SY5Y cells. APP is an integral membrane protein which undergoes post-translational modification such as glycosylation during its transfer through intracellular secretory pathway. The mature isoform of APP (i.e. APP holoprotein) is then acted upon by β- and γ-secretases to generate Aβ. The A2A receptor agonist, HENECA, induces production of Aβ42 in SH-SY5Y cells via cAMP/PKA/CREB pathway. It enhances both synthesis and maturation processes of APP increasing total APP production. It also stimulates γ-secretase activity in mAPP cleavage resulting in Aβ generation. H_2_S not only interferes with the step of APP maturation, but also attenuates the production of APP holoprotein. By inhibiting AC (and subsequent cAMP production), H_2_S also inhibits γ-secretase activity. It ultimately leads to decreased production in Aβ.

In a nutshell, current study suggests beneficial therapeutic role of H_2_S in AD as it interferes with HENECA stimulated Aβ42 production by attenuating APP maturation and inhibiting γ-secretase via a cAMP dependent pathway (Fig 9). By unraveling the underlying mechanisms of action, the inhibitory actions of H_2_S on adenosine A2A receptor signal transduction can be deeply studied and thus can be tweaked to gain the maximum therapeutic efficiency.
